# Mitophagy as an active regulator of cardiac metabolic reprogramming

**DOI:** 10.3389/fcvm.2026.1884607

**Published:** 2026-09-01

**Authors:** Li Gai, Yan Li, Chen Yang, Mengya Wang

**Affiliations:** Department of Cardiology, Karamay Central Hospital, Karamay, Xinjiang, China

**Keywords:** AMPK–ULK1, cardiovascular disease, FUNDC1, metabolic reprogramming, mitochondrial quality control, mitophagy, SIRT3–PGC-1*α*

## Abstract

Mitophagy is increasingly recognized as a context-dependent regulator of cardiac metabolic adaptation rather than solely as a disposal pathway for damaged mitochondria. By coupling mitochondrial turnover to substrate selection, redox control, and inflammatory signaling, mitophagy can influence fatty acid oxidation (FAO), glycolysis, and oxidative phosphorylation (OXPHOS) in cardiomyocytes, vascular endothelial cells, and immune cells. In this review, the term Mitophagy–Metabolic Rewiring Axis (MMRA) is used as an integrative conceptual framework—not as a newly discovered pathway or theory—to organize evidence for bidirectional interactions between mitophagy and metabolic remodeling. The framework comprises stress inputs, mitophagy machinery and flux, metabolic outputs, and cell- or disease-level consequences, while emphasizing that the biological effect of mitophagy depends on cell type, disease stage, and duration of activation. We critically assess the AMP-activated protein kinase (AMPK)–UNC-51-like kinase 1 (ULK1), sirtuin 3 (SIRT3)–peroxisome proliferator-activated receptor gamma coactivator 1-alpha (PGC-1*α*), PTEN-induced kinase 1 (PINK1)–Parkin E3 ubiquitin ligase, and hypoxia-inducible factor 1-alpha (HIF-1*α*)–BCL2-interacting protein 3 (BNIP3)/FUN14 domain-containing 1 (FUNDC1) modules in atherosclerosis, heart failure, and ischemia/reperfusion injury. Pharmacological, substrate-based, and exercise interventions are evaluated with particular attention to the predominantly preclinical evidence base, methodological limitations in measuring mitophagy flux, and the need for validated human biomarkers. Multi-omics and spatial approaches may improve mechanistic resolution, but clinical translation will require prospective studies that link target engagement to metabolic and cardiovascular outcomes.

## 1 Introduction

Cardiovascular disease (CVD) comprises heterogeneous disorders, including atherosclerosis, heart failure, and ischemia/reperfusion injury. Because the myocardium has a high and continuous energy demand, it is particularly vulnerable to hypoxic and oxidative stress. Nutritional and hemodynamic stress can redistribute metabolic flux among FAO, glycolysis, and the tricarboxylic acid cycle (1). In the short term, this remodeling may preserve ATP availability, whereas persistent metabolic deviation can promote energy deficiency, lactate accumulation, oxidative stress, mitochondrial injury, and cardiac dysfunction (2). Defining how mitochondrial quality control interacts with these metabolic transitions is therefore essential for distinguishing adaptive responses from progressive energetic failure.

**Figure 1:**
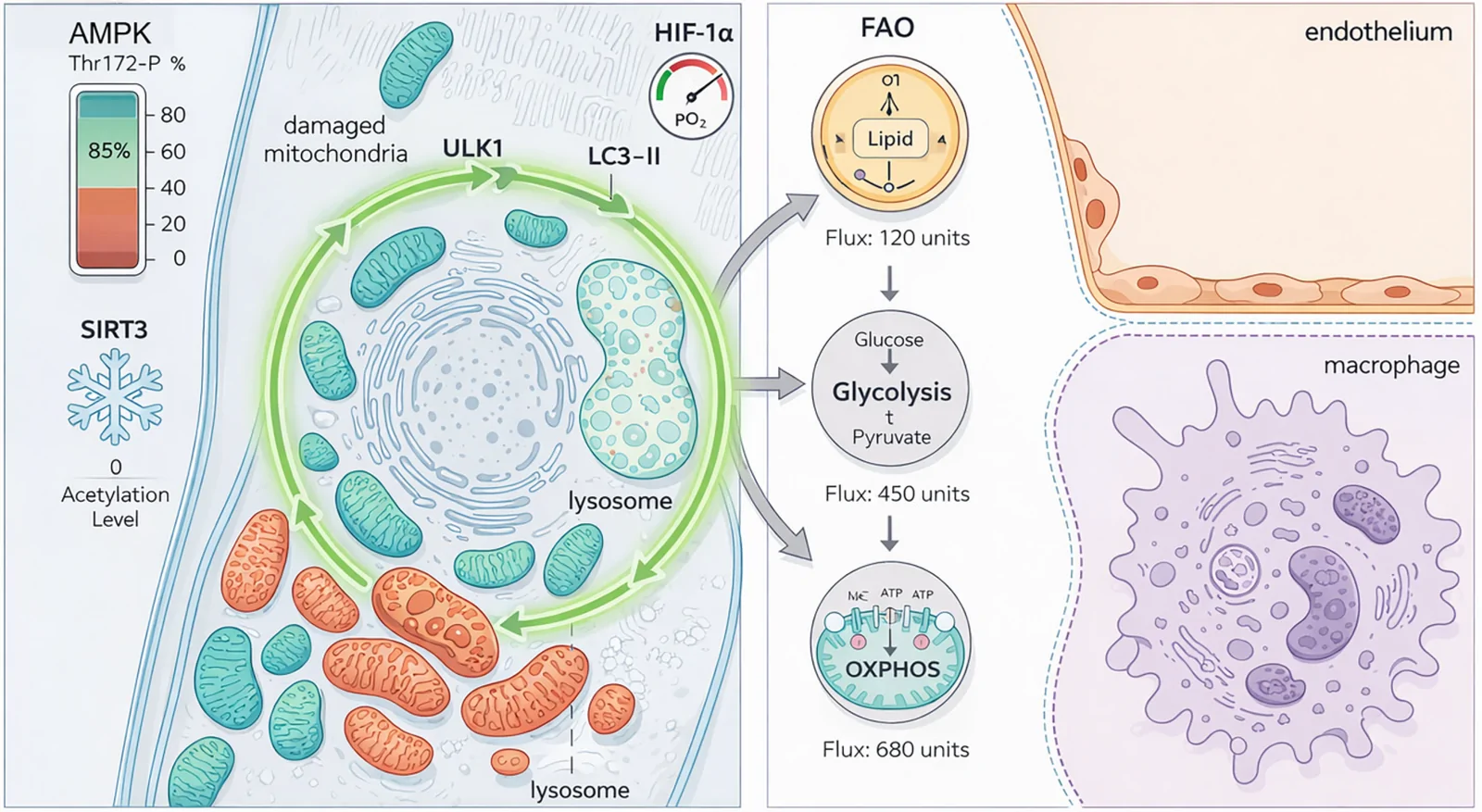
Schematic overview of mitophagy-associated metabolic reprogramming in cardiovascular cells. Energy- and hypoxia-responsive signals, including AMPK–ULK1, SIRT3, and HIF-1*α*, influence selective mitochondrial turnover and may thereby affect fatty acid oxidation (FAO), glycolysis, oxidative phosphorylation (OXPHOS), and cell-specific responses. The percentages and flux values are illustrative graphical scales rather than quantitative measurements, pooled estimates, or universally applicable thresholds.

Mitophagy selectively removes dysfunctional or superfluous mitochondria and thereby influences both the quality and size of the mitochondrial pool (3, 4). Its metabolic consequences extend beyond organelle clearance because mitochondrial turnover affects respiratory capacity, redox signaling, calcium handling, and substrate use. Major regulatory modules include PINK1/Parkin-dependent ubiquitination, receptor-mediated pathways involving BNIP3, NIX/BNIP3L, and FUNDC1, and energy-sensing pathways centered on AMPK, mTORC1, ULK1, and sirtuins. These modules do not operate as a single linear pathway, and their effects vary with cell type, disease stage, and stress duration. Accordingly, the MMRA is presented here as an integrative conceptual framework rather than a newly discovered theory. Figure 1 provides a simplified overview of this context-dependent relationship.

### 1.1 Operational definition, scope, and boundaries of the MMRA framework

For clarity, the MMRA is operationalized as four linked domains: stress/metabolic inputs → completed mitophagy flux → metabolic outputs → cell/disease consequences. We consider an MMRA claim strongest when a measured change in mitophagy flux is linked to a defined metabolic readout rather than inferred from a single marker.

The framework has explicit boundaries. It does not include all forms of macroautophagy, all mitochondrial dynamics, or every metabolic change observed in CVD. It does not assume that mitophagy is always upstream of metabolic remodeling, and it does not equate increased LC3-II or PINK1/Parkin expression with increased degradative flux. Basal mitophagy can also occur independently of PINK1 in tissues with high metabolic demand (5). The contribution of the MMRA is therefore an integrative synthesis that enables comparison of context, directionality, and evidence strength across studies.

Unlike prior cardiovascular reviews that mainly catalog mitophagy pathways, this review uses the MMRA to compare directionality, cell specificity, evidence strength, and translational relevance of mitophagy–metabolism coupling (6). Key gaps are whether pathway-marker increases represent productive flux or blocked degradation, when mitochondrial clearance shifts from adaptive to energetically costly, and whether metabolic remodeling is upstream, downstream, or parallel to altered mitophagy.

Under ischemic and oxidative stress, mitophagy can limit the persistence of ROS-generating mitochondria and support redox homeostasis (7). However, changes in PINK1/Parkin, AMPK–ULK1, or LC3-II abundance do not by themselves demonstrate completed mitophagy flux. Severe ROS accumulation can either trigger mitochondrial clearance or impair lysosomal completion, and the metabolic consequence of AMPK activation depends on timing and stress intensity (8, 9). Cellular context is equally important: FUNDC1-associated turnover has been linked to endothelial glycolytic support and barrier function (10), whereas impaired PINK1-related quality control in immune cells can accompany glycolytic and inflammatory activation (11). Mitophagy should therefore be interpreted as a context-dependent regulator of substrate use and stress adaptation rather than an invariably protective upstream switch.

Accordingly, this review compares PINK1/Parkin-, BNIP3/FUNDC1-, and AMPK–SIRT3–PGC-1*α*-related evidence across cardiomyocytes, endothelial cells, and immune cells, then examines atherosclerosis, heart failure, ischemia/reperfusion injury, and candidate interventions. Throughout, we distinguish marker-level association from validated flux, preclinical mechanism from human evidence, and conceptual therapeutic rationale from demonstrated cardiovascular benefit.

## 2 Signaling networks and dynamic regulation of mitophagy

Rather than cataloging every reported regulator, this section groups the evidence into three interacting modules: an energy-sensing and canonical quality-control module, a hypoxia-responsive receptor module, and a transcriptional or epigenetic modulatory layer. These modules converge on mitophagy flux but differ in trigger, cellular distribution, and downstream metabolic effect. Table 1 summarizes their principal components and reported metabolic implications.

**Table 1:** Signaling pathways and metabolic regulatory mechanisms of mitophagy in cardiovascular disease.

Signal transduction pathway	Main molecular composition	Key regulatory mechanism	Metabolic effects	Cardiovascular relevance	References
The canonical PINK1-Parkin pathway	PINK1, Parkin, ubiquitin, OPTN, NDP52	Upon mitochondrial membrane potential depolarization, PINK1 accumulates and phosphorylates both Parkin and ubiquitin, triggering K63-linked ubiquitination and subsequent recruitment of autophagy receptors.	Clears dysfunctional mitochondria and may support fatty acid oxidation (FAO) and tricarboxylic acid (TCA)-cycle efficiency.	PINK1 deficiency can reduce myocardial ATP availability, promote glycolytic compensation, and impair energetic efficiency.	(1, 5)
BNIP3/NIX non-canonical pathway	BNIP3, NIX, HIF-1*α*, LC3	Under hypoxic conditions, HIF-1*α* induces BNIP3/NIX expression, which mediates mitochondrial clearance via its LC3-interacting region (LIR).	Can restrict oxidative phosphorylation (OXPHOS) and favor glycolytic adaptation to hypoxia.	Insufficient signaling may impair stress adaptation, whereas excessive or prolonged activation can contribute to mitochondrial depletion.	(4, 12)
FUNDC1 oxygen-sensing pathway	FUNDC1, PGAM5, LC3, SIRT3	Under hypoxia, dephosphorylation of FUNDC1 by PGAM5 promotes LC3 binding and receptor-mediated mitophagy; SIRT3-related regulation has also been implicated in mitochondrial homeostasis.	Supports mitochondrial quality control, calcium homeostasis, and context-dependent metabolic adaptation.	FUNDC1 deficiency has been associated with impaired endothelial function, altered mitochondrial signaling, and energetic stress.	(13, 14)
The AMPK-mTOR-ULK1 energy-sensing axis	AMPK, mTORC1, ULK1, TSC2, Raptor	ATP depletion activates AMPK, which inhibits mTORC1 and phosphorylates ULK1 to initiate autophagy.	Promotes mitochondrial turnover and may support recovery of oxidative metabolism.	Contributes to myocardial energy homeostasis during ischemic stress, but prolonged activation may be maladaptive.	(8)
The SIRT1-SIRT3-PGC-1*α* acetylation-regulator*y* axis	SIRT1, SIRT3, PGC-1*α*, LKB1	SIRT1-mediated deacetylation of LKB1 can activate AMPK, whereas SIRT3 influences mitochondrial enzymes, redox control, and pathways linked to mitophagy and biogenesis.	Supports NAD+-dependent signaling, fatty acid oxidation, mitochondrial biogenesis, and antioxidant defense.	SIRT3 deficiency is associated with impaired cardiac metabolism; reported benefits of SIRT3 activation remain largely preclinical.	(9, 10)
The HIF-1*α*-BNIP3/FUNDC1 hypoxia-responsive axis	HIF-1*α*, BNIP3, FUNDC1, PHD2	Hypoxia stabilizes HIF-1α and can increase BNIP3- and FUNDC1-related mitophagy signaling.	May support short-term glycolytic compensation and hypoxia tolerance, with possible energetic costs during prolonged activation.	May be protective early in ischemic stress but detrimental when activation is excessive or clearance is incomplete.	(6, 15)
The miRNA/lncRNA-mediated epigenetic layer	miR-137, miR-351, H19, MALAT1, NEAT1	miRNAs can regulate mitophagy-related genes, whereas lncRNAs may modulate these networks through transcriptional, post-transcriptional, or competing endogenous RNA mechanisms.	Can alter autophagy-related gene expression and metabolic adaptation; direct evidence for completed mitophagy flux is often limited.	Reported effects are context dependent, and findings from individual miRNA–target pairs should not be generalized across disease models.	(16, 17)

Mitophagy is regulated by interacting oxygen-sensitive, energy-sensing, and transcriptional mechanisms rather than by a single hierarchical pathway (18). Under ischemic or hypoxic stress, BNIP3/NIX, FUNDC1, AMPK–ULK1, and non-coding RNA networks may converge on mitochondrial recognition and lysosomal turnover. The functional outcome depends on whether flux is completed and whether mitochondrial loss is matched by biogenesis and metabolic recovery.

Under hypoxia, BNIP3/NIX and FUNDC1 can recruit LC3 and promote receptor-mediated mitochondrial turnover (19). HIF-1*α*–BNIP3 signaling has also been linked to protection in non-cardiac ischemia/reperfusion models (12), while programmed mitophagy can facilitate a glycolytic switch during cell-state transitions (20). A recent editorial on preeclampsia underscores broader interest in mitochondrial stress pathways (21), but it does not provide direct mechanistic evidence for cardiac BNIP3-mediated mitophagy (21). In the heart, NIX has been associated with hypertrophy and cardiomyocyte death when excessively activated (22). FUNDC1 additionally regulates mitochondrial dynamics (14) and endoplasmic-reticulum–mitochondrial contacts (23), and FUNDC1-related signaling has been linked to mitochondrial integrity in obesity-associated cardiac dysfunction (24). Renal ischemia/reperfusion studies further support HIF-1*α*/FUNDC1-dependent mitophagy, although these findings should not be extrapolated directly to myocardium (25). Collectively, receptor-mediated mitophagy is best viewed as a context-dependent stress response rather than a uniformly protective mechanism.

Non-coding RNA regulation of cardiovascular autophagy is emerging but heterogeneous. The miR-212/132 family regulates cardiac hypertrophy and cardiomyocyte autophagy (16). miR-137 has been studied in non-cardiac hypoxia models, so its relevance to cardiac mitophagy remains uncertain (17). A recent critical-care review discusses ferroptosis and mitochondrial injury but does not establish a miR-351–FUNDC1 mechanism in myocardium (26). This source is therefore cited only as contextual evidence (26), not as direct support for a specific miR-351 pathway (26). By contrast, miR-23a has been linked to PGC-1*α*/Drp1-dependent mitochondrial injury in doxorubicin-treated cardiomyocytes (27).

Reviews indicate that non-coding RNAs can modulate cardiovascular autophagy, but many studies assess general autophagy rather than organelle-specific flux (28). MALAT1/miR-30a/Beclin-1 signaling has been examined in myocardial infarction (29). The same study does not establish NEAT1-mediated BNIP3 regulation (29), and evidence for HIF-1*α*/BNIP3-related autophagy in sepsis-induced myocardial dysfunction should be interpreted as a related, but distinct, mechanism (30). TUG1/miR-29a-3p has been associated with hypoxia-induced injury in AC16 cells (31). Broader epigenetic reviews emphasize the diversity and model dependence of these networks (32). Accordingly, non-coding RNA findings should be treated as pathway-specific observations rather than a unified mitophagy signature.

**Figure 2:**
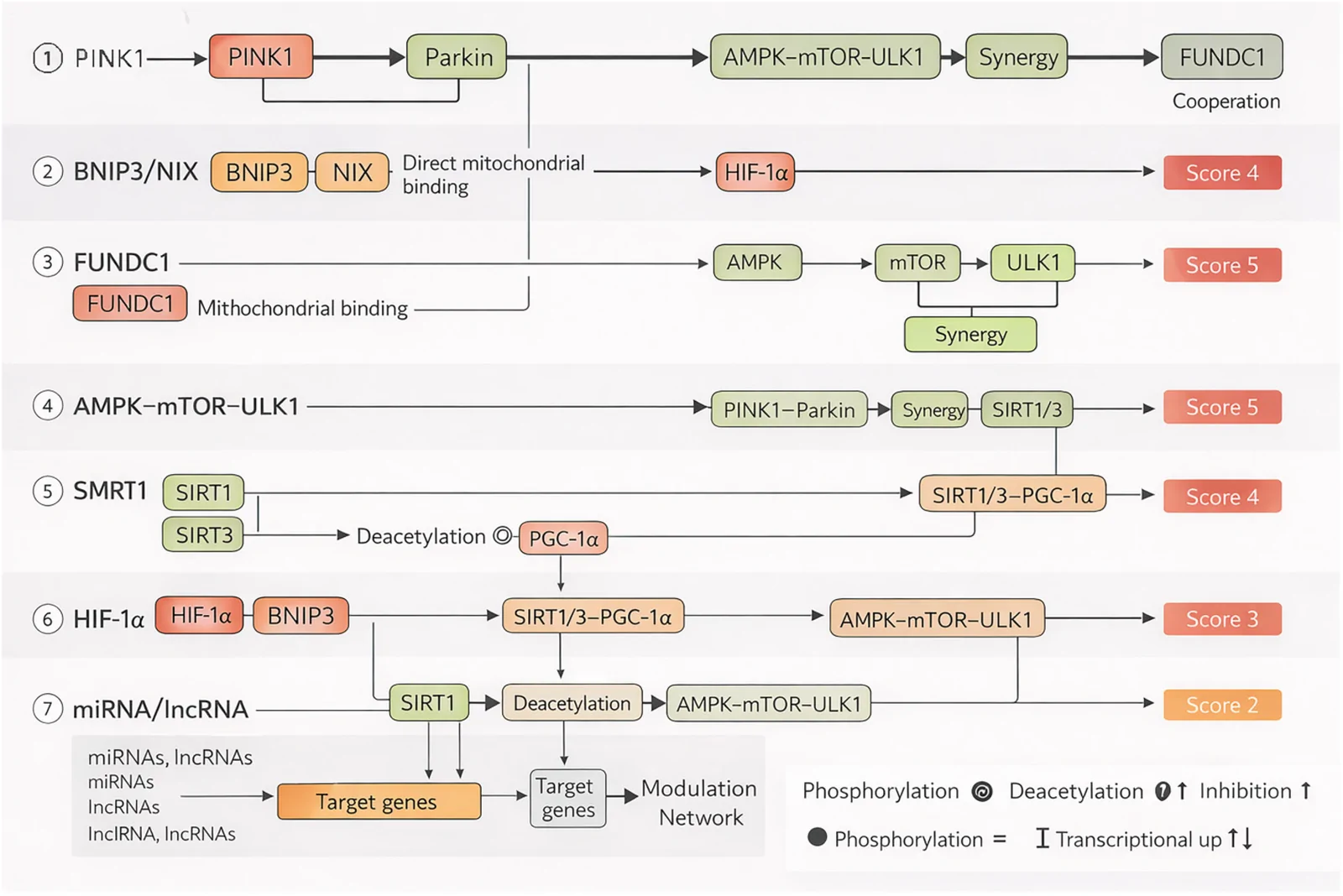
Schematic summary of regulatory modules connecting mitophagy to cardiovascular metabolism. The diagram assembles reported relationships involving PINK1–Parkin, BNIP3/NIX, FUNDC1, AMPK–mTOR–ULK1, SIRT1/3–PGC-1*α*, HIF-1*α*, and non-coding RNAs. Arrows, symbols, colors, and score labels are organizational devices rather than quantitative rankings or a single linear pathway; protein placement is schematic and not drawn to anatomical scale.

In summary, oxygen-responsive receptors and epigenetic regulators form interacting, context-dependent layers of mitophagy control. The evidence is strongest for specific molecular interactions and weakest when individual markers are generalized into a universal pathway. Figure 2 summarizes these modules and their reported relationships without implying a single linear signaling hierarchy.

A central methodological limitation is the distinction between autophagosome abundance and completed degradative flux. Increased LC3-II, p62/SQSTM1 accumulation, or mitochondria–lysosome colocalization may reflect enhanced initiation, impaired lysosomal clearance, or both. The ischemia/reperfusion literature demonstrates that autophagosome accumulation can coexist with defective clearance and cardiomyocyte injury (33). Robust interpretation therefore requires complementary flux assays, lysosomal controls, and metabolic measurements in the same experimental system.

## 3 Mitophagy-driven cardiovascular metabolic reprogramming

The three cardiovascular cell types considered here share a common logic—mitochondrial damage sensing, selective turnover, and downstream effects on redox and inflammatory signaling—but the metabolic variable being protected is different. Cardiomyocytes are constrained primarily by ATP-generating OXPHOS and FAO, endothelial cells by glycolytic support of barrier and angiogenic function, and macrophages by flexible coupling between mitochondrial quality control and inflammatory state. The subsections below therefore separate shared quality-control mechanisms from cell-specific metabolic endpoints rather than treating mitophagy as a uniform upstream switch.

### 3.1 Cardiomyocytes: mitophagy deficiency, energy depletion, and lipid accumulation

Mechanistic and disease-model studies have linked Parkin-mediated mitophagy to fatty-acid oxidation (34), SIRT3 to antihypertrophic mitochondrial defense (35), and Mst1-mediated SIRT3 suppression to impaired Parkin-dependent mitophagy in diabetic cardiomyopathy (36). These findings support cardiac relevance while remaining model specific.

Cardiomyocytes rely heavily on mitochondrial ATP production, so mitophagy must be coordinated with mitochondrial biogenesis and substrate supply. Loss of membrane potential or increased ROS can trigger selective mitochondrial turnover, but clearance alone does not regenerate the mitochondrial pool (37). The term “clearance–replacement cycle” is therefore more appropriate than implying that mitophagy directly induces new mitochondria. When FAO is constrained, glycolytic compensation may preserve ATP transiently, although sustained reliance on glycolysis can reduce energetic efficiency.

Genetic models show that impaired autophagy or Parkin-dependent quality control can promote mitochondrial dysfunction and abnormal lipid handling, but tissue-specific findings should not be transferred uncritically to myocardium (38). In the failing heart, increased reliance on the pyruvate–lactate axis may provide short-term compensation while contributing to metabolic remodeling (39). In dystrophic cardiomyopathy, reduced PINK1/Parkin-mediated mitophagy has been reported at late disease stages (40), whereas AMPK-dependent BCL2L13 signaling can support cardioprotective mitophagy (41). The latter evidence does not establish that AMPK activation is beneficial in every context (41). Together, these studies support a role for mitochondrial quality control in cardiac metabolism but do not justify a simple “more mitophagy is better” model.

SIRT3 and PGC-1*α* connect mitochondrial redox control, substrate oxidation, and biogenesis, and AMPK/SIRT1 signaling contributes to metabolic adaptation (42). However, the evidence does not support a single SIRT3–PGC-1*α* positive-feedback loop in all cardiac settings. General Atg7-deficiency studies illustrate the consequences of impaired autophagy but are not specific to mitophagy in the heart (43). HIF-1*α*/BNIP3 signaling can be protective during acute myocardial ischemia/reperfusion (44), whereas reviews of cardiovascular mitophagy emphasize that the direction of effect depends on timing, pathway, and flux completion (6). The same review evidence should be interpreted as synthesis rather than clinical validation (6).

### 3.2 Endothelial cells: FUNDC1, glycolytic dependence, and barrier function

Endothelial cells obtain most ATP from glycolysis, whereas mitochondria contribute disproportionately to signaling, ROS control, calcium handling, and stress responses. FUNDC1 is a hypoxia-responsive mitophagy receptor (13), but the original FUNDC1 study did not quantify endothelial glycolytic flux or establish a universal 30% reduction in glycolysis (13). Endothelial FUNDC1 also participates in metabolic signaling and SIRT3-related regulation (45). Other cardiovascular studies link mitophagy to reperfusion injury through distinct mechanisms (46) and show that PGAM5/CK2-dependent phosphorylation controls FUNDC1–LC3 binding (47). These data support a role for FUNDC1 in endothelial and cardiac stress responses, but they do not justify the conclusion that FUNDC1 alone determines endothelial glycolytic capacity.

PFKFB3-driven glycolysis is central to endothelial sprouting (48), while AMPK activation can influence vascular remodeling in other pharmacological contexts (49). These findings are compatible with, but do not directly prove, a FUNDC1–glycolysis axis. Claims that FUNDC1 activates SIRT3/MnSOD or that AMPK/HIF-1*α* activation fully reverses FUNDC1 deficiency should therefore be treated as hypotheses unless tested with endothelial-specific flux assays (47).

### 3.3 Macrophage immunometabolism: mitophagy and inflammatory states

Mitophagy contributes to macrophage metabolic remodeling and inflammatory signaling. Pro-inflammatory states often show greater glycolytic dependence, whereas reparative programs can rely more heavily on OXPHOS and FAO. The conventional M1/M2 classification is useful as a heuristic but does not capture the heterogeneous macrophage states observed in cardiovascular lesions. Mitophagy should therefore be discussed as one regulator of mitochondrial ROS, mtDNA release, inflammasome activation, and substrate use rather than as a binary switch between two fixed phenotypes.

Recent studies further show that MDM2 can coordinate inflammatory and glycolytic responses in M1-like macrophages (50), whereas PINK1/Parkin-dependent mitochondrial autophagy has been linked to altered macrophage polarization in a fluoride-exposure model (51). These models support mechanistic links but should not be generalized directly to cardiovascular lesions.

**Figure 3:**
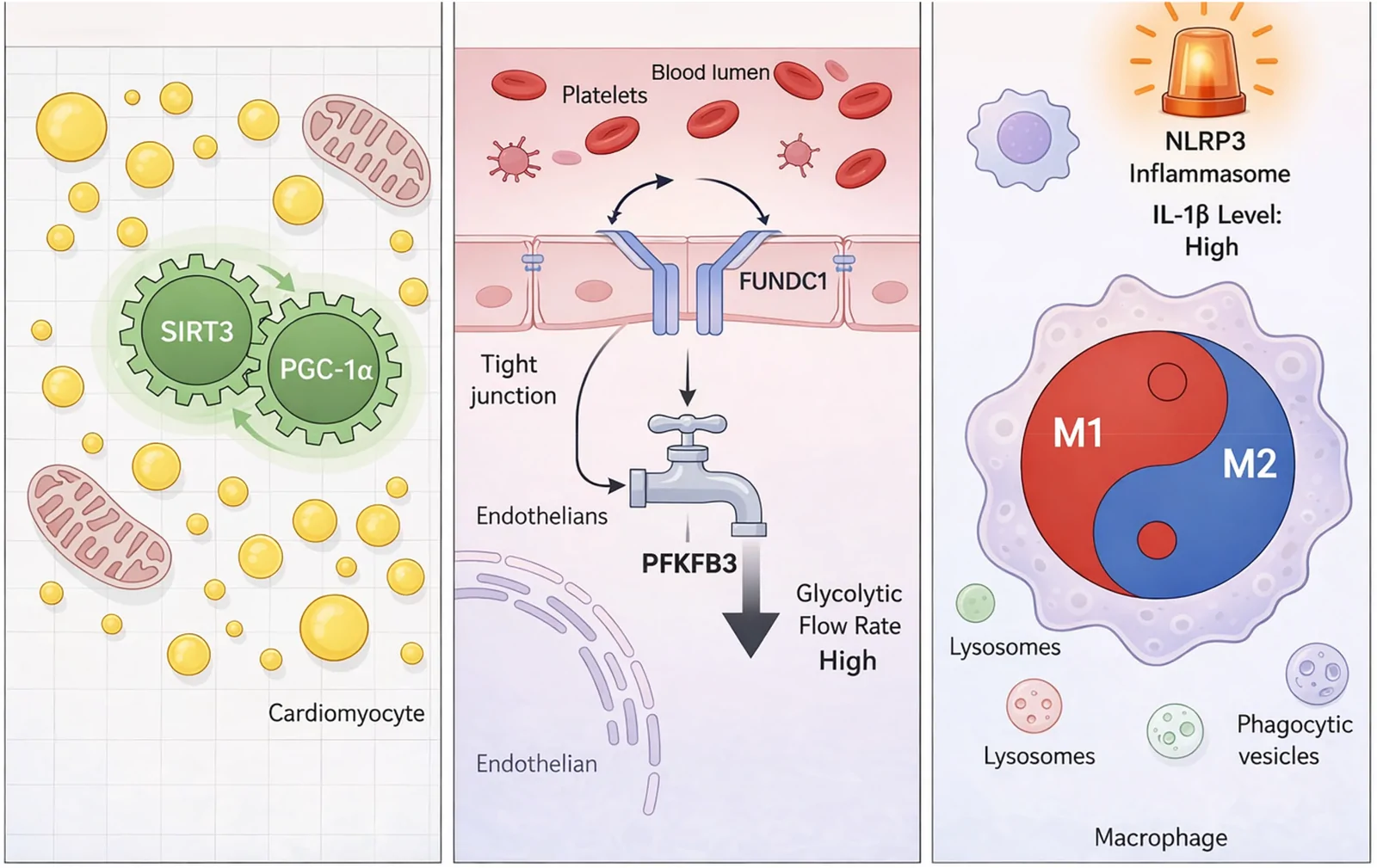
Cell-specific schematic of mitophagy–metabolism coupling in cardiomyocytes, endothelial cells, and macrophages. The panels summarize coordination of mitochondrial turnover and lipid handling in cardiomyocytes, FUNDC1-associated endothelial metabolic and barrier regulation, and the relationship between mitochondrial quality control, NLRP3 signaling, and macrophage functional states. The M1/M2 colors represent ends of a phenotypic continuum rather than mutually exclusive states, and molecular icons are schematic rather than spatially scaled.

AMPK–mTOR–ULK1 signaling can link energy stress to autophagy and immune metabolism (52). Mitochondrial quality control can restrain inflammasome activation by limiting damaged mitochondria (53), but direct evidence for a fixed SIRT3-dependent M1-to-M2 switch is limited. Reviews of mitochondrial dynamics emphasize that macrophage polarization spans a continuum rather than two discrete states (54). Mitochondrial dysfunction and mtDNA release can contribute to NLRP3 activation in some models (55), while single-cell studies demonstrate substantial macrophage heterogeneity in atherosclerotic lesions (56). Overall, mitophagy is one component of a broader immunometabolic network, and Figure 3 depicts phenotypic tendencies rather than binary states.

### 3.4 Cross-cell synthesis, evidence level, and methodological constraints

Across cardiomyocytes, endothelial cells, and macrophages, the shared mechanistic core is not a single molecular pathway but the coupling of mitochondrial damage recognition and lysosomal clearance to redox control, substrate handling, and inflammatory signaling. The dominant output differs by cell type: maintenance of respiratory reserve and lipid oxidation in cardiomyocytes, preservation of signaling and barrier function within a glycolysis-dominant endothelial program, and control of mtDNA/ROS-driven inflammatory metabolism in macrophages. Thus, the same increase in a mitophagy-related marker cannot be assigned the same metabolic meaning across cell types.

Evidence strength also differs substantially. Most causal data derive from genetic or pharmacological cell and animal models, whereas human studies mainly provide tissue expression, circulating biomarkers, imaging, or functional associations rather than direct measurements of cardiac or vascular mitophagy flux. Cross-study comparison is further limited by non-equivalent assays: static LC3-II, p62/SQSTM1, PINK1/Parkin abundance, or mitochondria–lysosome colocalization do not measure the same biological step as flux reporters with lysosomal controls; disease models differ in species, cell source, oxygen exposure, and acute versus chronic stress; and metabolic endpoints range from OCR/ECAR to isotope tracing, metabolomics, ATP content, or surrogate enzyme expression. These methodological differences can generate apparently conflicting conclusions and preclude quantitative comparison across studies. Future work should pair organelle-specific flux measurements with lysosomal blockade or validated reporters, mitochondrial mass, respiration, isotope-resolved substrate flux, and cell-specific functional outcomes in the same model before assigning clinical relevance (33, 57).

## 4 Disease-specific mitophagy–metabolic reprogramming

The relationship between mitophagy and metabolic remodeling differs across CVD phenotypes and disease stages. In atherosclerosis, macrophage and endothelial quality-control defects intersect with lipid handling and inflammation; in heart failure, the balance between mitochondrial clearance and biogenesis influences energetic reserve; and in ischemia/reperfusion injury, the effect of mitophagy changes over time as selective clearance can give way to impaired lysosomal completion or excessive mitochondrial loss. Table 2 summarizes these disease-specific patterns.

**Table 2:** Specific mechanisms of the mitophagy-metabolic reprogramming axis in different cardiovascular diseases.

Cardiovascular condition	Specific mechanisms	Metabolic effects	Related signaling pathways	References
Atherosclerosis	Impaired macrophage mitochondrial quality control contributes to inflammatory and metabolic imbalance.	Enhanced glycolysis, suppressed fatty acid oxidation, reduced cholesterol efflux, decreased ATP generation, and reactive oxygen species (ROS) accumulation	PINK1/Parkin, SIRT3, NF-*κ*B, NLRP3, AMPK-SIRT1-PGC-1*α*	(58–60)
Heart Failure	Imbalance between mitophagy and mitochondrial biogenesis contributes to energetic stress.	Reduced mitochondrial quantity, decreased oxidative phosphorylation efficiency, enhanced glycolysis, low energy efficiency, and suppressed fatty acid oxidation (FAO)	SIRT3-PINK1-Parkin, AMPK-PGC-1*α*, mTORC1	(61, 62)
Ischemia-Reperfusion Injury	Dysregulated initiation or incomplete clearance can contribute to mitochondrial loss, ROS accumulation, and metabolic failure.	Decreased fatty acid *β*-oxidation, lactic acid accumulation, TCA cycle blockage, compensatory enhancement of glycolysis, and reduced energy efficiency	AMPK-ULK1, BNIP3-Drp1, FUNDC1-SIRT3-AMPK	(33, 63, 64)

### 4.1 Atherosclerosis: mitochondrial quality control, inflammation, and metabolic imbalance

Atherosclerosis couples lipid retention, endothelial dysfunction, and inflammatory activation. Mitophagy may influence lesion biology through mitochondrial ROS, mtDNA release, and lipid handling, but direct causal evidence in plaque macrophages remains limited. Studies of cardiac lipid trafficking illustrate how altered acyl-CoA handling can drive pathological metabolic remodeling (65), while reviews of SIRT3 describe potential links among mitochondrial redox control, substrate metabolism, and inflammation (58). The same review evidence remains mechanistic rather than clinical (58). These observations are relevant to the MMRA framework but do not by themselves establish a macrophage-specific therapeutic axis.

FUNDC1 deficiency aggravates obesity and metabolic syndrome in mice, supporting a systemic role in mitochondrial quality control (59). However, this is not direct evidence that endothelial or macrophage FUNDC1 reverses atherosclerotic plaque biology. A review of the AMPK/SIRT1/PGC-1*α* axis in neurodegeneration illustrates broad metabolic conservation but is not cardiovascular-specific (60). Stronger mechanistic support for inflammasome coupling comes from studies showing that new mtDNA synthesis enables NLRP3 activation (66) and that autophagy proteins restrain mtDNA release (67). The mtDNA study also highlights the importance of distinguishing mitochondrial damage from productive mitophagy (66). Parkinsonism studies further demonstrate inflammation associated with PINK1/PRKN-related mitochondrial damage (68), but extrapolation to atherosclerosis requires plaque-specific validation. Overall, the evidence supports a plausible mitophagy–inflammation link rather than a clinically established therapeutic axis.

### 4.2 Heart failure: imbalance between mitochondrial clearance and biogenesis

Heart failure is characterized by reduced metabolic flexibility and impaired coordination among mitochondrial turnover, biogenesis, and substrate use. Evidence from non-cardiac systems shows that repression of PGC-1*α* can compromise mitochondrial programs (61) and that loss of membrane potential together with mTORC1 inhibition can trigger mitochondrial autophagy in mtDNA-mutant cells (69). These studies provide mechanistic context, but they do not establish a universal pattern of increased PINK1/LC3-II and decreased PGC-1*α* in human heart failure. Cardiac conclusions should therefore be based on direct myocardial flux and biogenesis measurements.

A SIRT3–PINK1–PKM2 axis has recently been described in osteoarthritis, where it linked mitochondrial renewal to metabolic switching (62). Because this evidence derives from cartilage rather than myocardium, it should not be presented as direct proof of SIRT3–PINK1 synergy in heart failure (62). It nonetheless illustrates a testable principle: mitochondrial clearance and metabolic reprogramming may need to be coordinated with biogenesis. Cardiac validation will require tissue-specific loss-of-function studies and simultaneous assessment of mitophagy flux, mitochondrial mass, respiration, and ventricular performance (62).

Mitochondrial dynamics and substrate handling may further modify heart-failure energetics. Drp1 is a central mediator of mitochondrial fission and apoptosis (70), while recent work has clarified systemic regulation of lactate homeostasis (71). AMPK–Drp1/PINK1–Parkin coupling has also been reported in non-cardiac metabolic disease (72). These studies support mechanistic plausibility but should not be interpreted as direct evidence that manipulating the same pathways will restore myocardial metabolism in heart failure.

Cardiac studies provide stronger disease relevance. BNIP3-mediated mitophagy requires Drp1-dependent fission and Parkin recruitment, and excessive signaling can impair bioenergetics (73). In failing human hearts, downregulation of PGC-1*α*/ERR*α* target genes is associated with impaired mitochondrial metabolic programs (74). Together, these findings support the concept that imbalance between mitochondrial removal and replacement may contribute to energetic failure, although direct longitudinal evidence in patients remains limited.

### 4.3 Ischemia/reperfusion injury: time-dependent protection and injury

In ischemia/reperfusion injury, the effect of mitophagy is strongly time dependent. Reviews emphasize that mitochondrial turnover can be homeostatic or pathogenic according to pathway, intensity, and duration (75). Experimental work has shown that autophagosome accumulation during reperfusion may reflect impaired clearance rather than productive flux (33). The same study demonstrated that defective autophagosome clearance contributes to cardiomyocyte death (33). Therefore, increases in LC3-II or autophagosome number should not be interpreted as beneficial mitophagy without lysosomal controls.

AMPK phosphorylation of ULK1 links energy stress to mitophagy initiation (63), whereas BNIP3 can impair bioenergetics while stimulating mitochondrial turnover (76). FUNDC1 activity is controlled by phosphorylation of its LC3-interacting region (77). SIRT3-related metabolic regulation has been demonstrated in renal fibrosis models (64), but direct relevance to cardiac ischemia/reperfusion requires confirmation. Melatonin studies provide preclinical evidence that AMPK–OPA1 signaling and mitochondrial quality control can reduce ischemia/reperfusion injury (78). Collectively, these data support a time-sensitive protective-window hypothesis rather than a universally beneficial intervention.

Stable-isotope approaches can resolve changes in cardiac substrate flux during disease (57). Mitochondrial ATP suppression can also provide a signal for NLRP3 activation (79), linking energetic failure to inflammatory signaling. However, reduced fatty acid oxidation, lactate accumulation, ROS production, and impaired autophagosome–lysosome fusion should not be combined into a single linear mechanism unless measured in the same model. The key unresolved question is whether inflammatory activation follows defective mitophagy, precedes it, or emerges in parallel from mitochondrial injury.

Corosolic acid has also been reported to attenuate cardiac ischemia/reperfusion injury through a PHB2/PINK1/Parkin-related mitophagy pathway in a preclinical model (80), providing additional mechanistic evidence but not clinical validation.

**Figure 4:**
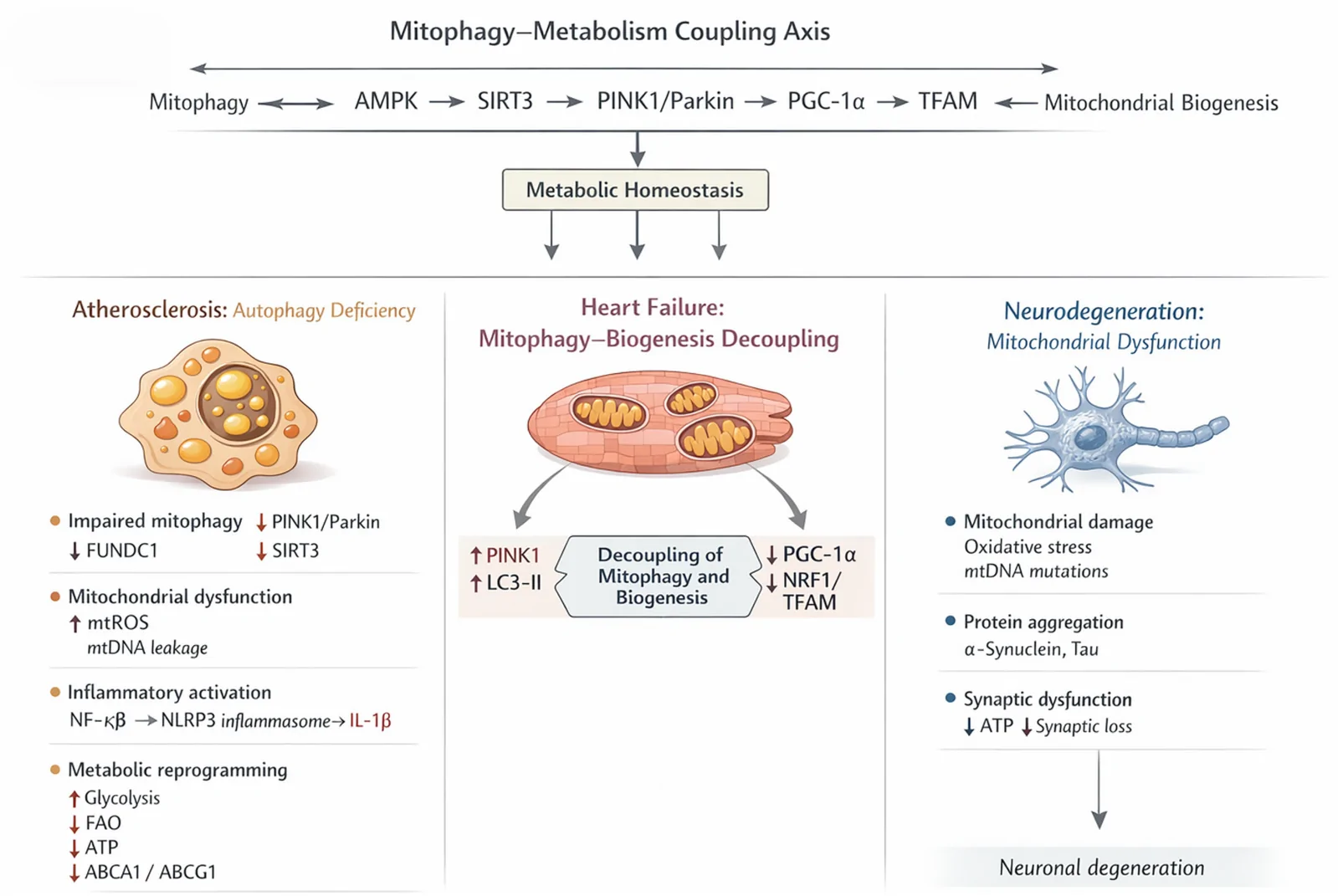
Comparative schematic of disrupted mitophagy–metabolism coupling in atherosclerosis, heart failure, and a non-cardiovascular neurodegeneration example. The cardiovascular panels illustrate how impaired mitochondrial clearance or uncoupling from mitochondrial biogenesis may contribute to inflammatory-metabolic imbalance and energetic failure. The neurodegeneration panel is included only as a cross-system comparison of conserved mitochondrial consequences and is not used as evidence for cardiovascular disease mechanisms.

Available clinical observations are mainly associative and do not establish that higher PINK1/Parkin expression represents greater protective mitophagy. In ischemia/reperfusion injury, the functional effect depends on timing, selectivity, and completion of flux: moderate early clearance may limit ROS-generating mitochondria, whereas persistent activation, blocked lysosomal degradation, or non-selective mitochondrial loss may worsen energy failure. Accordingly, AMPK–SIRT3–FUNDC1-related interventions should be evaluated against direct measures of flux, mitochondrial mass, respiration, and cardiac function. Figure 4 summarizes this context-dependent interpretation.

## 5 Therapeutic modulation of mitophagy and metabolic reprogramming

Therapeutic modulation of mitophagy–metabolism coupling can be approached through small molecules, metabolic regulators, combined interventions, and exercise. The central translational challenge is that improvement in a pathway marker is not equivalent to completed mitophagy flux or clinical benefit. Most interventions discussed below have been evaluated predominantly in cells or animal models, and their cardiovascular effects may arise from mechanisms beyond mitophagy. The evidence is therefore considered according to target engagement, flux validation, metabolic outcome, functional outcome, and level of clinical support.

### 5.1 Small-molecule modulators: urolithin A, spermidine, and nicotinamide riboside

Urolithin A, spermidine, and nicotinamide riboside are discussed because they influence mitochondrial turnover, NAD+-dependent signaling, or metabolic capacity. They are not equivalent “MMRA activators,” and evidence ranges from cell and animal studies to limited human biomarker trials. The following assessment separates mechanistic plausibility from demonstrated cardiovascular efficacy.

Urolithin A is a gut microbial metabolite of ellagitannins. Preclinical and early human studies suggest effects on mitochondrial quality and cardiovascular biomarkers (81). In acute cardiac injury associated with severe pancreatitis, urolithin A improved cardiomyocyte fatty-acid oxidative metabolism in a preclinical model (82), but this does not establish efficacy in chronic heart failure (82). Foundational work showed that urolithin A induces mitophagy and improves muscle function in worms and rodents (83). A randomized trial in middle-aged adults reported improvements in muscle performance and mitochondrial-health biomarkers, not direct cardiac mitophagy (84). Thus, urolithin A is promising but remains clinically unproven as a cardiovascular mitophagy therapy.

Spermidine influences autophagy through pathways including eIF5A hypusination and TFEB translation (85), while SIRT3 regulates mitochondrial fatty-acid oxidation through reversible deacetylation (86). In aged mice, spermidine improved cardiac function and lifespan-related outcomes (87), but that study did not establish the proposed p53–BNIP3 mechanism or isolate mitophagy as the sole mediator (87). Additional work links spermidine to improved mitochondrial biogenesis and cardiac aging (88), and higher dietary intake has been associated with lower mortality in an observational cohort (89). These findings support further investigation but do not demonstrate causal cardiovascular efficacy in humans.

Nicotinamide riboside (NR) increases NAD + availability and can enhance oxidative metabolism in preclinical models (90). In a small trial of heart failure with reduced ejection fraction, NR was reported to be safe and tolerable; the study was not designed to prove improvements in ATP synthesis, mitochondrial membrane proteins, or clinical outcomes (91). Accordingly, the same trial should not be cited as evidence for the specific percentage changes originally stated (91). NR can activate SIRT3 in other organ systems (15), and NAD + repletion improves mitochondrial and stem-cell function in mice (92), but direct evidence that NR restores cardiac mitophagy flux in patients remains limited.

Together, urolithin A, spermidine, and nicotinamide riboside provide mechanistically plausible ways to influence mitochondrial turnover, NAD+-dependent signaling, and metabolic capacity. However, their effects are pleiotropic, human cardiac mitophagy has rarely been measured directly, and clinical cardiovascular efficacy has not been established. Future studies should measure completed flux, respiration, substrate oxidation, mitochondrial mass, and cardiac function in the same experimental system.

### 5.2 Metabolic regulators: AICAR, trimetazidine, and ketone-based approaches

AICAR is an experimental AMP mimetic widely used to probe AMPK signaling, but it also has AMPK-independent effects on mitochondrial oxidative phosphorylation (93). Exercise studies show that AMPK phosphorylation of ULK1 is required for targeting mitochondria to lysosomes in skeletal muscle (94), not that a single AICAR dose doubles cardiac mitophagy or improves myocardial oxidative phosphorylation by a fixed percentage (94). AMPK and SIRT1 cooperate in skeletal-muscle adaptation to fasting and exercise (42), but this is not direct evidence for a cardiac AICAR–SIRT3–PGC-1*α* pathway (42). Contemporary reviews further emphasize that AMPK can either promote or restrain autophagy depending on context (95). AICAR should therefore be considered a mechanistic tool rather than a clinically validated cardiovascular therapy.

Trimetazidine shifts myocardial substrate use from fatty acid oxidation toward glucose oxidation by inhibiting long-chain 3-ketoacyl-CoA thiolase (96). Meta-analytic evidence suggests potential functional benefit in heart failure with reduced ejection fraction, although study quality and heterogeneity limit certainty (97). References describing hepatic malonyl-CoA metabolism (98) or AMPK/ACC/CPT1 activation by tea extracts in obese mice (99) do not directly demonstrate trimetazidine-induced mitophagy. In diabetic cardiomyopathy models, trimetazidine reduced fibrosis and apoptosis and enhanced general autophagy (100), but the study did not establish PINK1/Parkin-dependent mitophagy as the causal mediator (100).

Ketone bodies provide an alternative cardiac fuel and may influence signaling beyond substrate oxidation. Intravenous *β*-hydroxybutyrate acutely increased cardiac output in patients with chronic heart failure (101), but the study did not establish reduced ROS or enhanced ATP synthesis as the mechanism (101). Evidence that *β*-hydroxybutyrate suppresses NLRP3 activation comes partly from acute kidney injury models (102), and mTORC1–TFEB regulation is a general autophagy mechanism rather than ketone-specific proof (103). A randomized trial of oral ketone ester in heart failure with reduced ejection fraction provides emerging clinical evidence (104), but effects on cardiac mitophagy flux remain unmeasured.

AICAR, trimetazidine, and ketone-based therapy affect different metabolic targets and should not be presented as interchangeable activators of one pathway. AICAR is primarily an experimental AMPK-modulating tool, trimetazidine alters substrate selection, and ketone bodies provide an alternative fuel. Their effects on mitophagy remain context dependent and, in several cases, indirect. A rational MMRA-based strategy should match the intervention to the dominant metabolic defect and verify both metabolic correction and mitochondrial quality-control responses.

### 5.3 Exercise as a physiological modulator of mitophagy and cardiac metabolism

Exercise is a physiologically relevant intervention that repeatedly perturbs energy demand, redox state, substrate use, and mitochondrial turnover. Acute exercise can activate AMPK–ULK1 signaling, whereas training can promote coordinated mitochondrial biogenesis, dynamics, and quality control (42, 94). Within the MMRA framework, exercise is therefore not simply another “activator” but a systems-level stimulus that tests whether mitophagy and metabolic remodeling remain appropriately coupled.

Exercise-related evidence supports improved mitochondrial health and functional capacity, but mechanistic interpretation requires caution. Human trials have often measured skeletal-muscle performance or circulating mitochondrial biomarkers rather than mitophagy flux in the heart (84). Exercise benefits also arise from hemodynamic, endothelial, skeletal-muscle, autonomic, and anti-inflammatory adaptations. Exercise mode, intensity, duration, disease stage, and recovery interval may therefore produce different cardiac mitochondrial responses.

### 5.4 Combined strategies: coordinating mitochondrial turnover and metabolic support

Combining a mitophagy-modulating intervention with metabolic support is conceptually attractive because clearance of damaged mitochondria must be accompanied by adequate substrate availability, respiratory recovery, and mitochondrial replacement. However, proposed combinations remain mainly mechanistic or preclinical, and pathway convergence does not by itself establish therapeutic synergy.

Direct evidence for combining AICAR with urolithin A in cardiovascular disease is currently lacking. The AMPK–ULK1 exercise study supports a role for energy sensing in mitochondrial targeting (94), but it did not test AICAR plus urolithin A or demonstrate synergistic improvements in myocardial ATP, TCA-cycle flux, or contractility. Such a combination should therefore be presented as a hypothesis for future testing.

AMPK/ULK1-dependent phosphorylation of Parkin provides a mechanistic basis for pathway convergence (105). Separately, SIRT3–AMPK-related mitochondrial biogenesis has been studied in sepsis-induced myocardial injury (106). These findings do not constitute evidence that combined AICAR and NR therapy synchronizes mitophagy and biogenesis in cardiac ischemia/reperfusion. Any proposed combination should be evaluated with factorial designs, target-engagement assays, flux measurements, and functional endpoints.

Likewise, no cited study directly demonstrates a combined spermidine–ketone therapy in murine heart failure. Endothelial FUNDC1–SIRT3 signaling provides relevant mechanistic context (45), while spermidine reviews summarize autophagy and geroprotection (107). Those reviews do not establish synergy with ketone bodies (107). Manipulation of the NAD+/NADH ratio can support mitochondrial electron transport in experimental systems (108), but this should not be interpreted as evidence for a 45% increase or improved ejection fraction from the proposed combination.

The cited literature also does not directly test AICAR plus trimetazidine or AMPK activation plus ketone therapy. AMPK/ULK1-dependent Parkin phosphorylation (105) and AMPK-mediated mitochondrial fission during energy stress (109) provide a rationale for studying combination strategies, but not proof of therapeutic synergy. Future studies should compare monotherapy and combination arms and assess toxicity, mitochondrial mass, completed mitophagy flux, substrate oxidation, and cardiac function.

**Figure 5:**
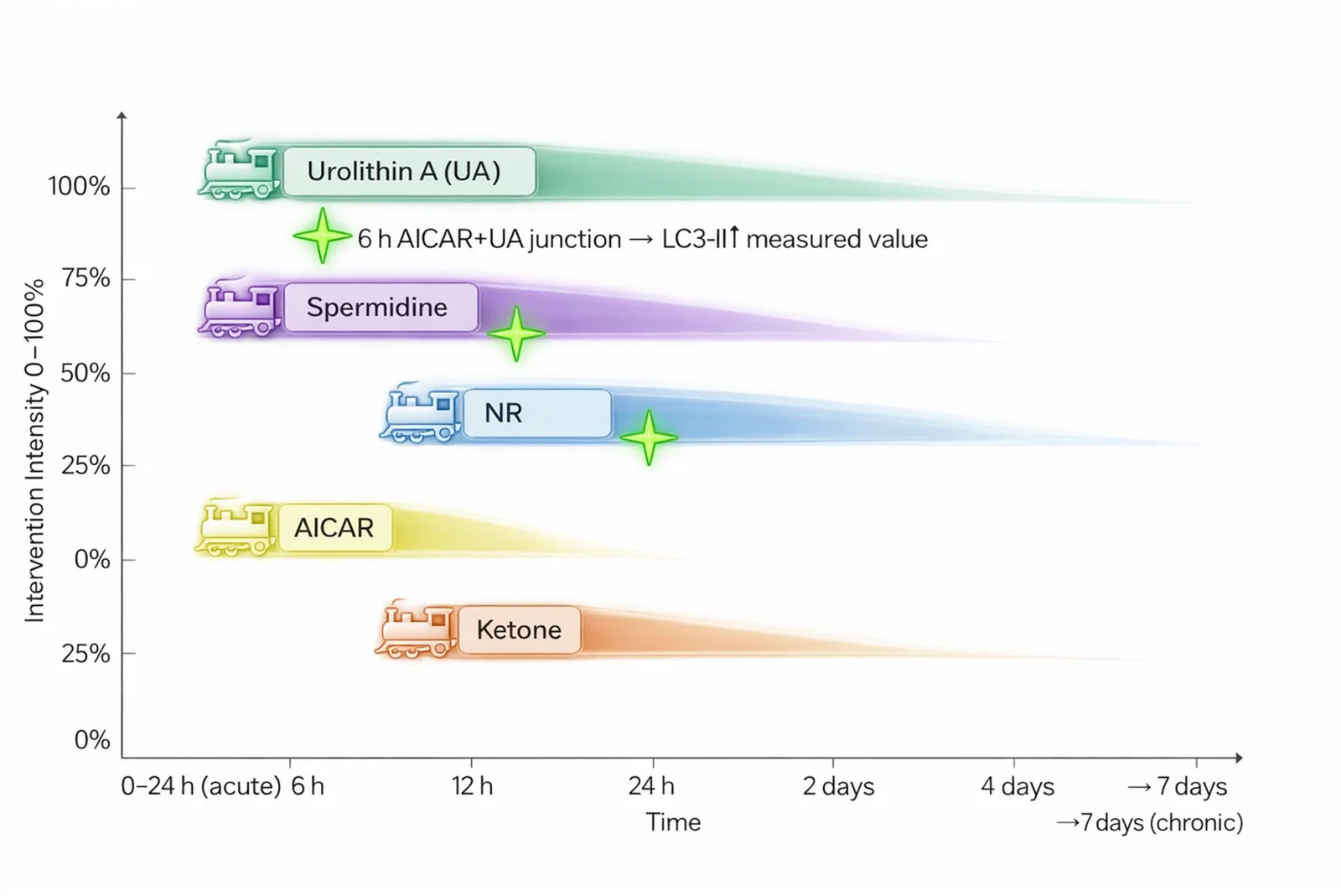
Conceptual comparison of interventions discussed in relation to mitophagy and metabolic remodeling. Urolithin A, spermidine, nicotinamide riboside (NR), AICAR, and ketone-based approaches are displayed as distinct intervention classes with different proposed targets and evidence bases. The time axis, percentages, graphical trajectories, and star markers are illustrative design elements and do not represent head-to-head efficacy, validated dose–response relationships, or experimentally measured treatment kinetics.

Timing is likely to be as important as target selection. During acute ischemic stress, transient enhancement of selective clearance may reduce ROS-generating mitochondria; during recovery, mitochondrial biogenesis and substrate oxidation may become more important. In chronic heart failure, prolonged non-selective activation could reduce energetic reserve. This temporal model is a testable hypothesis rather than an established treatment sequence (110, 111). Figure 5 presents the intervention classes as a conceptual comparison; the displayed trajectories and intervention intensities are illustrative rather than quantitative.

## 6 Future perspectives

**Figure 6:**
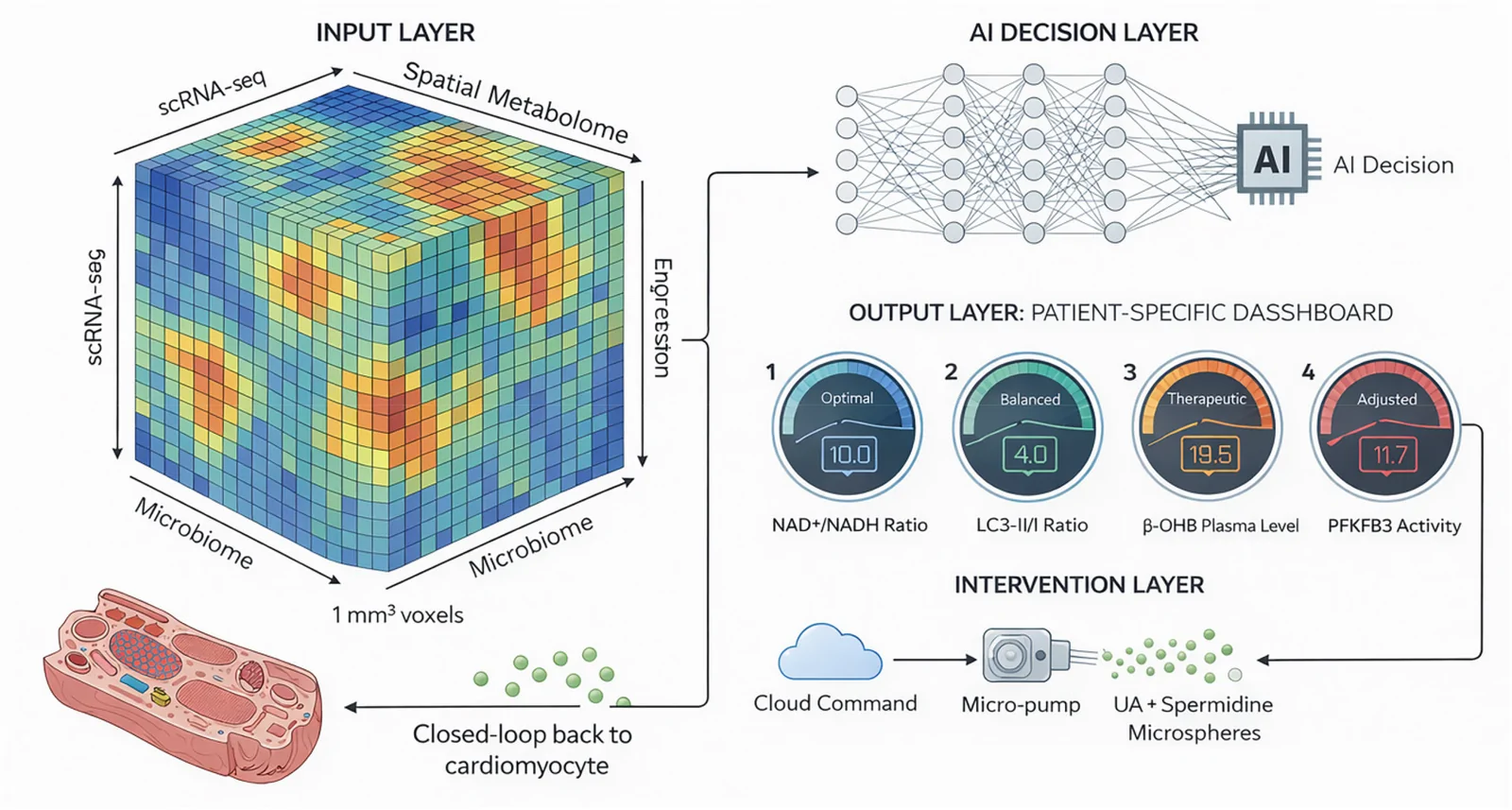
Conceptual research workflow for integrating multi-omics, spatial data, imaging, and computational analysis in studies of mitophagy–metabolism coupling. The diagram illustrates a hypothesis-generating decision-support framework linking data acquisition, pattern analysis, candidate biomarker interpretation, and prospective intervention testing. Dashboard values, voxel dimensions, and delivery components are schematic examples rather than validated clinical thresholds or an autonomous treatment system.

Future progress will depend on improving causal and translational resolution rather than adding increasingly complex pathway diagrams. Studies should quantify completed mitophagy flux with organelle-specific reporters and lysosomal controls while measuring respiration, isotope-resolved substrate use, mitochondrial mass, and cardiovascular function in the same experiment. Cell-specific and spatial approaches are needed to distinguish cardiomyocyte, endothelial, fibroblast, and immune-cell responses within diseased myocardium, and human studies require validated biomarkers linked to imaging and clinical outcomes. Multi-omics and machine-learning methods may help identify response patterns, but they should be presented as analytical tools rather than autonomous treatment systems. The MMRA framework will be useful only if it remains falsifiable, distinguishes association from causation, and accommodates evidence that the same mitophagy pathway can be protective, neutral, or harmful according to context. Figure 6 outlines this research workflow.
